# Impact of AKI care bundles on kidney and patient outcomes in hospitalized patients: a systematic review and meta-analysis

**DOI:** 10.1186/s12882-021-02534-4

**Published:** 2021-10-08

**Authors:** Hannah A. I. Schaubroeck, Diana Vargas, Wim Vandenberghe, Eric A. J. Hoste

**Affiliations:** 1 Intensive Care Unit, Ghent University Hospital, Ghent University, C. Heymanslaan 10, 9000 Ghent, Belgium; 2 Department of Nephrology, Saint Ignacio University Hospital, Bogota, Colombia; 3 Research Foundation-Flanders (FWO), Brussels, Belgium

**Keywords:** Acute kidney injury, Care bundle, Meta-analysis, Prevention, Systematic review

## Abstract

**Background:**

A bundle of preventive measures can be taken to avoid acute kidney injury (AKI) or progression of AKI. We performed a systematic review and meta-analysis to evaluate the compliance to AKI care bundles in hospitalized patients and its impact on kidney and patient outcomes.

**Methods:**

Randomized controlled trials, observational and interventional studies were included. Studied outcomes were care bundle compliance, occurrence of AKI and moderate-severe AKI, use of kidney replacement therapy (KRT), kidney recovery, mortality (ICU, in-hospital and 30-day) and length-of-stay (ICU, hospital). The search engines PubMed, Embase and Google Scholar were used (January 1, 2012 - June 30, 2021). Meta-analysis was performed with the Mantel Haenszel test (risk ratio) and inverse variance (mean difference). Bias was assessed by the Cochrane risk of bias tool (RCT) and the NIH study quality tool (non-RCT).

**Results:**

We included 23 papers of which 13 were used for quantitative analysis (4 RCT and 9 non-randomized studies with 25,776 patients and 30,276 AKI episodes). Six were performed in ICU setting. The number of trials pooled per outcome was low. There was a high variability in care bundle compliance (8 to 100%). Moderate-severe AKI was less frequent after bundle implementation [RR 0.78, 95%CI 0.62–0.97]. AKI occurrence and KRT use did not differ between the groups [resp RR 0.90, 95%CI 0.76–1.05; RR 0.67, 95%CI 0.38–1.19]. In-hospital and 30-day mortality was lower in AKI patients exposed to a care bundle [resp RR 0.81, 95%CI 0.73–0.90, RR 0.95 95%CI 0.90–0.99]; this could not be confirmed by randomized trials. Hospital length-of-stay was similar in both groups [MD -0.65, 95%CI -1.40,0.09].

**Conclusion:**

This systematic review and meta-analysis shows that implementation of AKI care bundles in hospitalized patients reduces moderate-severe AKI. This result is mainly driven by studies performed in ICU setting. Lack of data and heterogeneity in study design impede drawing firm conclusions about patient outcomes. Moreover, compliance to AKI care bundles in hospitalized patients is highly variable. Additional research in targeted patient groups at risk for moderate-severe AKI with correct and complete implementation of a feasible, well-tailored AKI care bundle is warranted. (CRD42020207523).

**Supplementary Information:**

The online version contains supplementary material available at 10.1186/s12882-021-02534-4.

## Introduction

Acute Kidney Injury (AKI) occurs in 7 to 18% of hospital admissions and 57% of intensive care unit (ICU) admissions [1, 2]. AKI is associated with increased length of hospital stay, morbidity and mortality. Moreover, increasing severity of AKI is associated with worse prognosis [1]. The excess hospital costs due to AKI consist of 3 to 14,000 $ per admission [3].

For the diagnosis of AKI the KDIGO working group classified AKI according to changes in serum creatinine level compared to baseline creatinine and/or urinary output [4]. To identify patients at risk for AKI, specific AKI biomarkers such as the cell cycle arrest biomarkers tissue inhibitor of metalloproteinase-2 and insulin-like growth factor-binding protein 7 (measured as TIMP-2*IGFBP7), neutrophil gelatinase associated lipocalin (NGAL), or chitinase 3-like protein 1 (CHI3L1) can be used [5–7].

There is no specific treatment targeting AKI. However, a bundle of preventive measures can be taken to avoid AKI or progression of AKI as described in the KDIGO guidelines. These include the avoidance of nephrotoxic agents and optimization of fluid status and hemodynamics [4, 8].

A care bundle can be defined as “a structured method of improving processes of care and patient outcomes; a small, straight-forward set of evidence-based practices, treatments and/or interventions for a defined patient segment or population and care setting that, when implemented collectively, significantly improves the reliability of care and patient outcomes beyond that expected when implemented individually” [9]. Given large variation in care for AKI patients and poor outcomes of AKI, the interest in implementing care bundles for AKI is growing. This bundle can consist of an e-alert for AKI, fluid balance and volume assessment, diagnostic tests with urine dipstick and echography, medication adjustment, avoidance of nephrotoxic agents, follow-up by a nephrologist and escalation of therapy or palliative care if necessary [9].

We aim to study the compliance to AKI care bundles in hospitalized patients and the impact of its application on kidney and patient outcomes by performing a systematic review and meta-analysis of existing literature.

## Materials and methods

### Study design

We conducted a systematic review and meta-analysis according to the PRISMA guidelines (Supplementary Table 1) [10, 11]. The protocol was registered in the PROSPERO database (CRD42020207523).

### Eligibility criteria

We included randomized controlled trials (RCT’s), retrospective and prospective observational, propensity-matched or intervention studies on the implementation of a care bundle for AKI. The studied population were adult and paediatric patients (ICU, emergency department, medical and surgical wards) with AKI or at risk of AKI during hospitalization.

Only articles published in English, Dutch, Spanish and French were included in this meta-analysis. Articles on AKI in outpatient setting and primary care were excluded as well as articles concerning care bundles on sepsis, liver cirrhosis, resuscitation, or other bundles in which AKI was not the main focus. Case reports, reviews, editorials, intervention studies evaluating a specific treatment, duplicate publications and articles which did not report on the outcome measures were excluded. The KDIGO practice guideline for AKI recommended the concept of a specific AKI bundle in 2012. Therefore, we included studies published since 2012 [4].

### Outcomes and prioritization

The review was restricted to studies that report compliance of care bundles, kidney outcomes such as occurrence of AKI and moderate to severe AKI (KDIGO stage 2 and 3), in-hospital use of kidney replacement therapy (KRT) and kidney recovery and patient outcomes (ICU, in-hospital and 30-day all-cause mortality and ICU and hospital length-of-stay or LOS).

### Search strategy

The first selection of the search was performed by one investigator (D.V.), under supervision of the principal investigator (E.A.J.H.), who is a content expert. A second independent search was performed by a third investigator (H.S.). The scientific search engines PubMed, Embase and Google Scholar were used. The search included the period January 1, 2012 till June 30, 2021. The bibliographies of relevant papers were consulted to retrieve potentially relevant citations. First, D.V. consulted Pubmed with following key words: “(acute kidney injury OR acute tubule necrosis)AND(bundle OR care bundle OR compliance). OR quality OR assessment quality OR AKI bundle)” and MeSH Terms (“patient compliance AND patient care bundles AND (acute kidney injury OR kidney tubular necrosis)”). In Web of Science we used the search terms (‘acute kidney failure’/exp. OR ‘acute kidney failure’ OR ‘acute kidney tubule necrosis’/exp. OR ‘acute kidney tubule necrosis’). In Google Scholar “acute kidney injury care bundles” and “acute kidney injury care bundle” were searched.

A second independent search was performed by H.S. in PubMed using the high-performance search filters (sensitivity) for AKI as described by Hildebrand et al. [12], combined with the following terms: (“patient care bundles”[MeSH Terms] OR (“patient”[All Fields] AND “care”[All Fields] AND “bundles”[All Fields]) OR “patient care bundles”[All Fields] OR (“care”[All Fields]) AND “bundle”[All Fields]) OR “care bundle”[All Fields]. To search Web of Science the following search terms were combined: ‘acute kidney injury OR acute kidney failure OR acute renal failure OR acute tubule necrosis’ and ‘care bundle’ OR ‘bundle’. In Google Scholar “acute kidney injury” and care bundle was used. Last search was performed 30th of June, 2021. The full-text articles were screened by D.V. and H.S. for further eligibility. The agreement between D.V. and H.S. was substantial (Cohen’s ƙ = 0,75). In case of discussion (9 articles), E.H. made the final decision.

Citations of included papers were collected using Endnote X9 (Clarivate®).

### Data extraction and collection

The collected data were directly extracted to an Excel database (Microsoft 2013®). Data extracted from each study included first author, year of publication, study period, country, study design, sex and age of study participants, outcomes, elements of the care bundle, and elements for assessment of quality of included studies. Data were extracted independently by two reviewers (D.V. and H. S.) using a data extraction form.

### Data synthesis and quality assessment

Papers containing raw data on a predetermined outcome were included in the quantitative analysis and pooled by outcome. Per outcome, studies were clustered by design (RCT’s versus before-after intervention studies) and by setting (all settings vs ICU setting only).

For statistical analysis the software program SPSS Statistics 26 (IBM Corporation and Others®) was used. The meta-analysis was performed with the software package Review Manager (RevMan) version 5.4.1 (Copenhagen: The Nordic Cochrane Centre, The Cochrane Collaboration, 2020), using the Mantel Haenszel test (risk ratio, reported with 95% confidence interval) and inverse variance (mean difference, 95% confidence interval) for length-of-stay. A random effect model was used to combine the data due to the expected diversity in methodology and clinical approach used in the included studies. Heterogeneity was assessed using a forest plot and the I^2^ statistic [13]. As a sensitivity analysis we analysed the randomized controlled trials separately. Bias was assessed by the risk of bias tool that is available in RevMan for the RCT’s and with the NIH study quality tool for non-randomized studies. (https://www.nhlbi.nih.gov/health-topics/study-quality-assessment-tools) (Supplementary Table 2). A funnel plot was constructed for the assessment of publication bias.

## Results

### Study selection

**Fig. 1:**
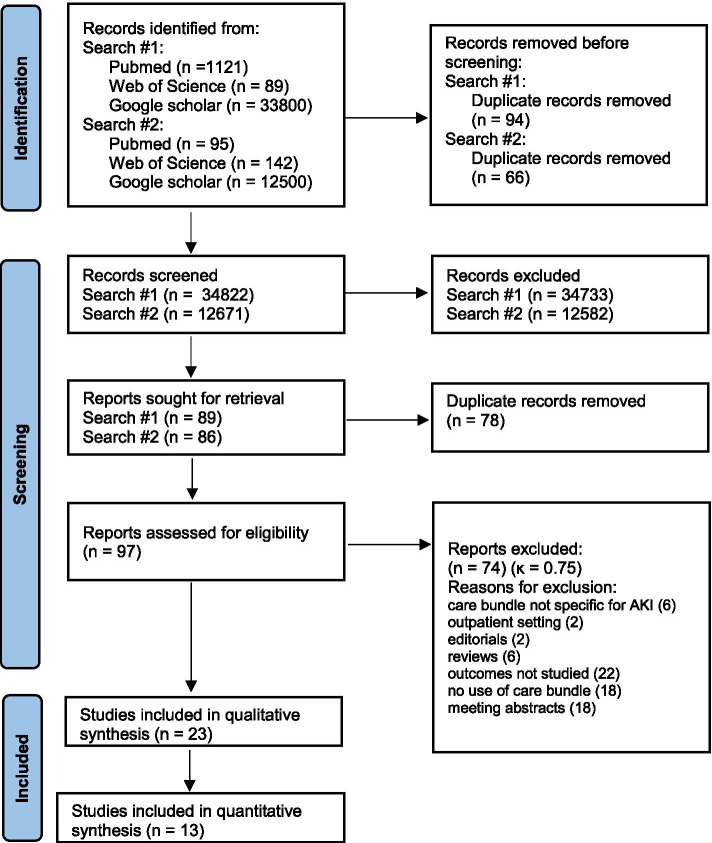
PRISMA 2020 flow diagram of study selection

Search results are presented in the PRISMA flow chart (Fig. 1). Following identification and screening of the results, the systematic literature search yielded 97 potential studies. The final analysis included 23 papers (40,874 patients and 30,276 AKI episodes) containing data on implementation of AKI care bundles of which 13 for quantitative analysis [8, 14–35]. Design of the studies used for quantitative analysis were RCT (4), retrospective before after study (5), propensity-matched before after study (1), prospective before after interventional study (2) and stepped wedge cluster randomized trial (1). Three RCT’s and 3 before after studies included only ICU patients.

### Baseline characteristics and care bundle compliance (Fig. 2)

Baseline characteristics of the included studies are shown in Table 1. The content of the used AKI care bundles varied largely; components of each bundle are described in Table 2. The KDIGO bundle was most frequently used [8, 20, 22, 26, 30, 32, 35], often in association with biomarker-guided strategies. There was a high variability in overall compliance to the care bundle, ranging from 8 to 100%.

**Fig. 2:**
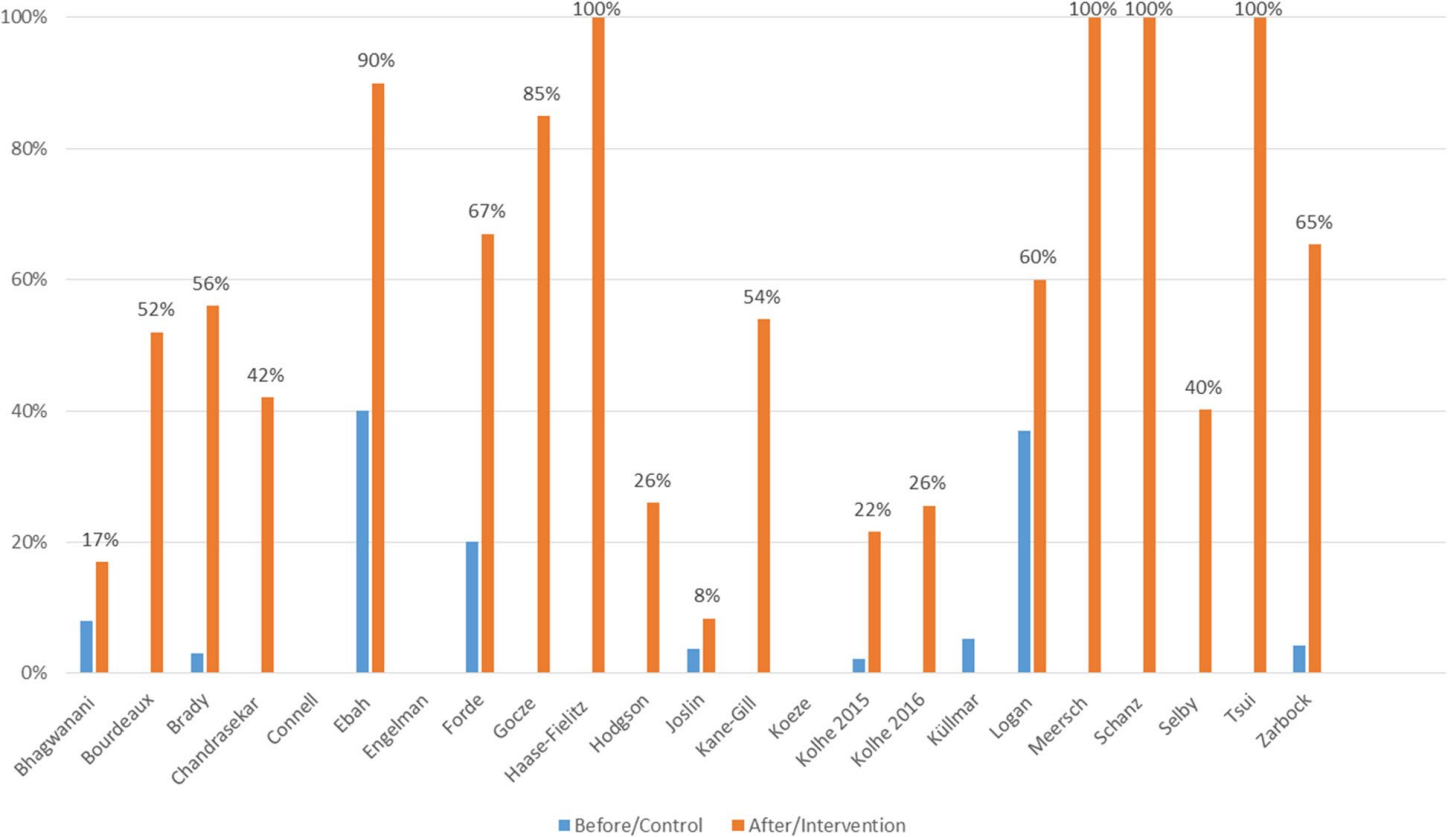
Compliance to AKI care bundles in all included studies; Before: a bundle of measures were reviewed without routine implementation of a care bundle; After/Intervention: a care bundle was structurally implemented

### Outcomes

All studied outcomes are summarized in Table 3. The number of trials pooled per outcome was generally low.

#### Occurrence of AKI in general, moderate-severe AKI and use of kidney replacement therapy (KRT) (Fig. 3)

Overall, there is a reduction in moderate-severe AKI when patients were treated with a care bundle; occurrence of AKI in general or use of KRT were not significantly decreased by implementation of a care bundle. The overall pooled risk ratio for occurrence of AKI is 0.90 [95%CI 0.76–1.05], for moderate-severe AKI 0.78 [95%CI 0.62–0.97] and for KRT 0.67 [95%CI 0.38–1.19].

**Fig. 3:**
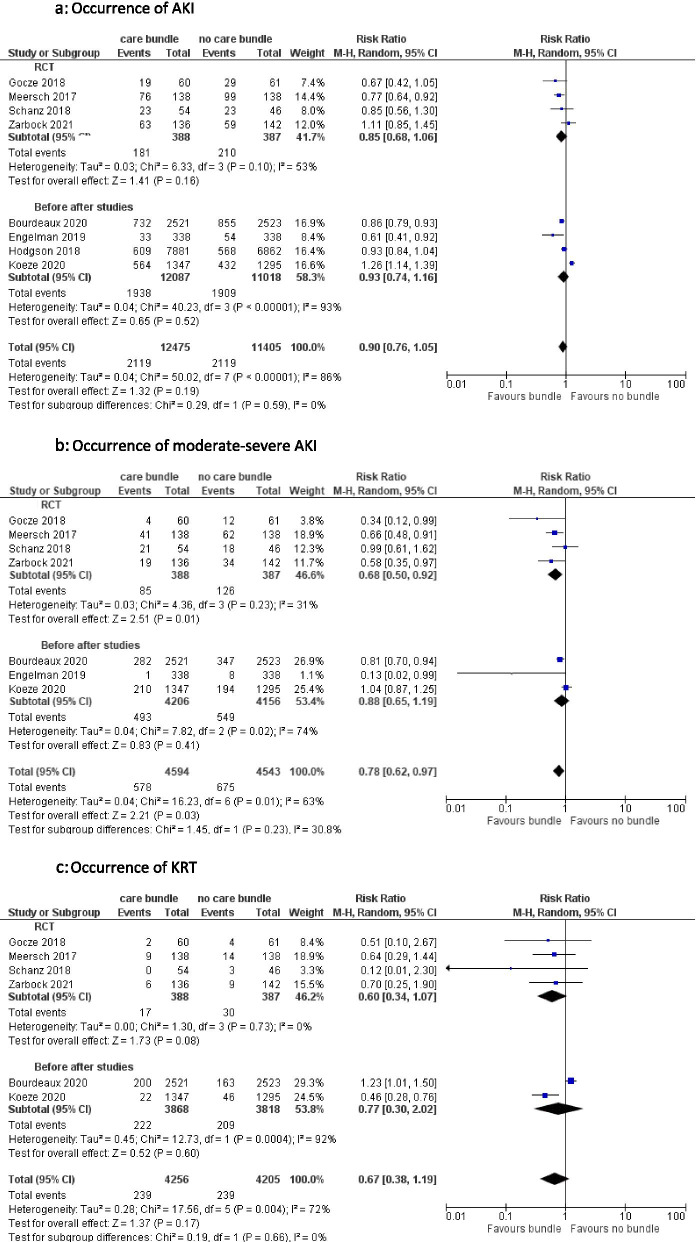
**a** Occurrence of AKI. **b** Occurrence of moderate-severe AKI. **c** Occurrence of KRT

Pooled analysis of 4 RCT’s (775 patients) showed a non-significant decrease in AKI occurrence [RR 0.85, 95%CI 0.68–1.06] with moderate heterogeneity between results, a significant decrease in moderate-severe AKI when a care bundle was implemented [RR 0.68, 95%CI 0.50–0.92] with low heterogeneity and a non-significant decrease in use of KRT [RR 0.60, 95%CI 0.34–1.07] without significant heterogeneity.

The decrease in occurrence of AKI in general [RR 0.93, 95%CI 0.74–1.16], moderate-severe AKI [RR 0.88, 95%CI 0.65–1.19] or use of KRT [RR 0.77, 95%CI 0.30–2.02] in patients treated with or without a care bundle was non-significant when pooling the outcomes of the before after studies. Heterogeneity between these studies was moderate or high. Similar results were obtained when pooling results from 2 studies that looked at AKI episodes instead of patients. (Supplementary Fig. S1) [8, 15, 20, 22, 24, 27–29, 32, 34–36].

When limiting the analysis to studies with ICU patients only, these findings were confirmed. Occurrence of moderate-severe AKI was markedly lower in the group treated with a care bundle when pooling the RCT’s [RR 0.62, 95%CI 0.47–0.80] and reached also significance in the overall analysis [RR 0.74, 95%CI 0.58–0.96] [8, 15, 20, 22, 27, 35]. (Supplementary Fig. S2).

#### All-cause ICU mortality, in-hospital mortality and 30-day mortality (Fig. 4)

There was a wide range of reported all-cause in-hospital mortality amongst the included studies, varying from 1.8 to 27% [22, 24, 25, 27–29, 32, 34]. Pooled data from studies examining in-hospital mortality in all patients, did not show a significant difference between the two groups [RR 1.03, 95%CI 0.73–1.46], with low heterogeneity. The 2 RCT’s that studied in-hospital mortality showed a non-significant decrease in patients exposed to a care bundle [RR 0.60, 95%CI 0.24–1.50]. Pooling the before after studies demonstrated a lower in-hospital mortality after care bundle implementation in AKI patients [RR 0.81, 95%CI 0.73–0.90]. Heterogeneity was not significant.

**Fig. 4:**
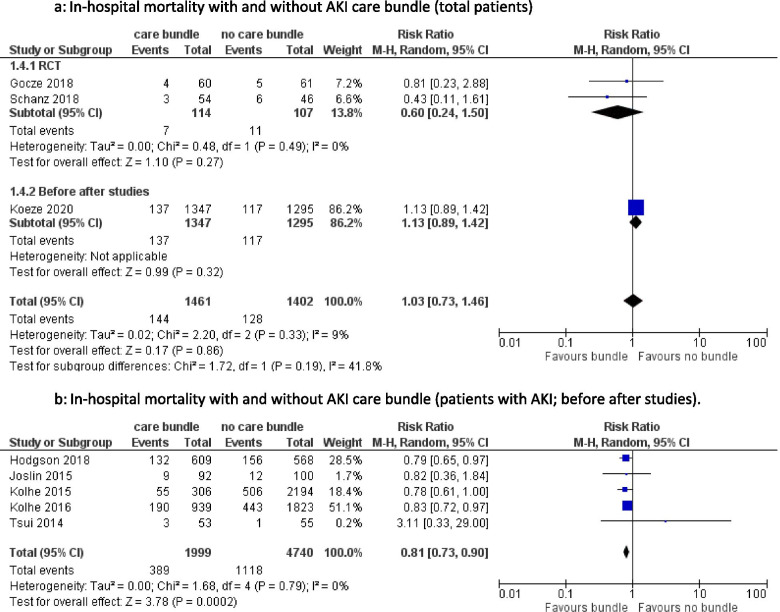
**a** In-hospital mortality with and without AKI care bundle (total patients). **b** In-hospital mortality with and without AKI care bundle (patients with AKI; before after studies)

ICU mortality in all patients was studied by Koeze and Bourdeaux [15, 27]. There was no significant difference between the control and the intervention group [RR 0.94, 95%CI 0.58–1.53]. Heterogeneity was moderate. Only 4 studies reported on 30-day all-cause mortality. Meersch et al. (RCT) and Engelman et al. (before after design) found no significant difference in 30-day mortality between all patients treated with or without care bundle [RR 1.00, 95%CI 0.42–2.39] [8, 20]. Pooled data from two before-after studies reached significance in decrease in 30-day mortality in patients with AKI and implementation of a care bundle [RR 0.95 95%CI 0.90–0.99] [29, 33]. (Supplementary Fig. S3).

#### Hospital and ICU length-of-stay (LOS) (supplementary fig. S4)

The hospital LOS was not significantly shorter when an AKI care bundle was implemented in 3 pooled before after studies [mean difference − 0.65, 95%CI -1.40,0.09] [28, 29, 37]. Since 10 studies reported hospital LOS as median with interquartile ranges (IQR) due to lack of normal distribution, they could not be included in this analysis [38]. Four studies provided data on ICU length-of-stay (3 reported as median and IQR, 1 as mean with standard deviation); only Gocze at al. found a significant shorter ICU LOS after care bundle implementation (median difference 1 (0,2) day; *p* = 0.035) [8, 15, 22, 27].

#### Kidney recovery

Three studies evaluated kidney recovery. Connell et al. only reported that the odds ratio for kidney recovery (defined as return to creatinine level within 120% of baseline prior to hospital discharge) was not different between patients treated with versus without care bundle [OR 1.03, 95% CI 0.56–1.87] [18]. Time to kidney recovery was shorter in patients with intervention compared to patients without intervention [OR 1.04, 95%CI 1.00–1.08]. Haase-Fielitz et al. performed a small randomized controlled study of 52 patients and found kidney recovery (defined as return to baseline creatinine at discharge) in 50% of patients who had intensified treatment with a care bundle versus 42% without care bundle, however this was not significant [OR 1.4, 95%CI 0.5–4,0] [23]. Zarbock and colleagues found a non-significant increase in kidney recovery in patients treated with a care bundle [OR 0.72; 95%CI 0.40–1.31] [35].

### Risk of bias analysis

In the RCT’s, performance bias was present. There was a low risk of detection, attrition and reporting bias. In the non-randomized trials, bias due to deviations from intended interventions was generally high and blinding of interventions was not feasible. (Supplementary Table 3) Funnel plots were constructed (Supplementary Fig. 5). Due to the low numbers of studies per outcome, publication bias could not be assessed.

## Discussion

In this systematic review and meta-analysis on AKI care bundles in hospitalized patients a total of 23 studies were included. Thirteen studies, consisting of 25,776 patients (of which 775 patients in 4 RCT) and 30,276 AKI episodes, were used for quantitative analysis. The overall compliance to all aspects of the AKI care bundle were highly variable between studies and rather low. Compliance to the bundle was higher in the RCT’s, ranging from 65 to 100%. This meta-analysis demonstrates that the occurrence of AKI was not significantly decreased in patients exposed to an AKI care bundle compared to standard of care. Importantly, occurrence of more severe stages of AKI, which is known to be associated with mortality [1, 2], was less frequent after implementation of a bundle. The use of KRT did not differ between the intervention and control group. In-hospital mortality and 30-day mortality was found to be lower in AKI patients exposed to a care bundle; this is solely based on non-randomized trials. A benefit on hospital and 30-day mortality could not be confirmed by RCT’s. Heterogeneity was not important for these outcomes. Only a limited number of studies could be pooled for hospital length-of-stay which did not show a difference between groups.

### Care bundle compliance

The overall compliance to all aspects of the bundle were highly variable and depended partially on study design and the included patient population. The randomized controlled trials had higher care bundle compliance (which is inherent to the design of the study), as well as the trials performed in ICU setting. In this setting, more health care staff is available which allows more rigorous follow-up compared to a regular hospital ward.

In 2012, the KDIGO working group made recommendations to implement an AKI care bundle routinely to reduce postoperative AKI in high-risk patients [4]. Küllmar and colleagues investigated the compliance with the KDIGO recommendations in routine clinical practice and found low compliance (5.3%) [30]. Compliance to the bundle could be improved by using an interruptive alert which demands to complete an electronic care bundle [29, 39]. A prospective singe centre cohort study showed that this strategy resulted in increased early interventions in ICU patients [40]. However a meta-analysis on interruptive e-alert implementation for detection of AKI could not show benefit on outcomes [41]. A possible explanation is ‘bundle or alert fatigue’, due to the already high administrative burden and the high number of items that needed to be checked [31]. Clinical decision support systems may help to recognize AKI and implement care bundle measures in an early phase. A concise and practical bundle is crucial for timely bundle completion. According to findings by Kolhe and colleagues, care bundle completion within 24 h after identifying AKI was associated with lower in-hospital mortality and less progression of AKI to higher stages [29].

### Occurrence of AKI, moderate-severe AKI and use of KRT

Moderate and severe AKI was less frequent in patients exposed to an AKI care bundle, in contrast with the occurrence of AKI in general. There could be several explanations for this finding. A higher detection rate of AKI due to increased awareness for AKI might be responsible for the absence of reduction of all stages of AKI after care bundle implementation [33]. Level of care may play a role as well as the clinical context of AKI. Three out of 4 RCT’s were performed in ICU setting only which all showed reduction in moderate-severe AKI [8, 22, 35]. Two retrospective before after studies found similar results. Engelman et al. observed a decrease in moderate-severe AKI in ICU patients post cardiac surgery and Bourdeaux et al. observed a lower proportion of increase from stage 1 to stage 2 or 3 after AKI care bundle implementation in a mixed ICU (42% vs 33.5%, *p* = 0.002) [15, 20]. Koeze et al. could however not demonstrate any benefit of care bundle implementation in a mixed ICU population [15, 20, 27]. The latter did not report on compliance to the used care bundle. Appropriate patient selection could be the key to success of AKI care bundle implementation. The BigpAK [22], PrevAKI single centre [8], PrevAKI multicentre RCT [35] and one interventional study by Engelman et al. [20] identified patients at risk for AKI with urinary biomarkers. They all showed a significant reduction in occurrence of moderate-severe AKI after AKI care bundle implementation. In contrast, they could not uniformly demonstrate an effect on AKI occurrence in general or on long-term patient outcomes. Moreover, 3 of these trials were performed in a cardiosurgical ICU only and applied the care bundle proposed by the KDIGO working group [4]. This could imply that using this care bundle is particularly beneficial in patients at risk for AKI in a specific context (e.g. ICU patients post major surgery).

### ICU/in hospital/30-day mortality – hospital length-of-stay (LOS)

In-hospital mortality was lower in AKI patients with an AKI care bundle. A potential risk of bias might be that better follow-up of patients exposed to a care bundle leads to lower hospital mortality. Other studies, not included in the quantitative analysis due to lack of raw data, confirmed this reduction in hospital mortality after implementation of an AKI care bundle [17, 19, 42]. This reduction on patient outcomes could not be demonstrated by randomized data which could be due to the small patient numbers included in the RCT’s and the variability in studied outcomes. The hospital length-of-stay was not significantly reduced in patients exposed to a care bundle, however this should be interpreted with caution because of the low number of included studies. We would expect a beneficial effect of a care bundle on the prognosis of the patient when there is reduction in AKI severity since AKI, and particularly moderate-severe AKI, is associated with increased mortality [1, 2]. A large, sufficiently powered RCT is warranted to investigate hard patient outcomes such as 30 day mortality.

### Limitations and strengths

First, the variability in components of the applied care bundles and compliance to the care bundle in the included studies was high, which makes it hard to draw conclusions about the effects of implementation of AKI care bundles in general. Secondly, we observed heterogeneity in study design, studied patient population and bundle implementation. For example, some investigators studied patients with AKI at time of inclusion, while other trials included patients without AKI. The use of AKI episodes instead of number of patients with AKI made comparison between studies difficult. High between-study heterogeneity in comparing AKI occurrence made interpretation of the results unclear. Third, several trials studied the implementation of AKI care bundles without collection of data on hard kidney and patient outcomes or did not report raw data and could therefore not be included in the final analysis. Fourth, our main results were driven by trials performed in an ICU setting, so we should be careful to extrapolate these findings to all hospitalized patients.

To our knowledge, this is the first systematic review and meta-analysis that reports kidney and patient outcomes related to AKI care bundle implementation. It was performed according to the PRISMA guidelines and preregistered in the Prospero database. It is unclear whether AKI care bundle implementation is in general beneficial, however this analysis raises some questions to address in future research, such as the best design of an AKI care bundle in terms of compliance, feasibility and prevention of ‘bundle fatigue’ as well as appropriate selection of patient groups, for example ICU patients in a specific context or screened for AKI risk by biomarkers who might benefit most of AKI care bundle implementation.

## Conclusion

This systematic review and meta-analysis shows that implementation of AKI care bundles does not influence the occurrence of AKI in general. In contrast, the occurrence of moderate to severe AKI is reduced after care bundle implementation. Lack of data and heterogeneity in study design and results impede drawing firm conclusions about patient outcomes. Compliance to AKI care bundles in hospitalized patients is highly variable. Additional research in targeted patient groups at risk for AKI with correct and complete implementation of a feasible AKI care bundle, well-tailored and integrated in the pre-existing clinical support system, could help to clarify the impact of its implementation on kidney and patient outcomes.

## Supplementary Information

**Additional file 1: Figure S1**: Occurrence of moderate-severe AKI and KRT (AKI episodes). **Figure S2**: Occurrence of AKI, moderate-severe AKI and KRT in ICU patients only. **Figure S3**: ICU and 30 day mortality. **Figure S4**: Hospital length-of-stay (AKI episodes).

**Additional file 2: Figure S5**: Funnel plots of main outcomes.

**Additional file 3: Table TS1**: PRISMA 2020 Checklist for conducting a systematic review and meta-analysis.

**Additional file 4: Table TS2**: Risk of bias tool for non-randomized trials.

**Additional file 5: Table TS3**: Risk of bias analysis.

## Data Availability

Additional data are available on request by contact with the corresponding author (H.S.).

## Abbreviations

AKI: acute kidney injury
ICU: intensive care unit
KDIGO: Kidney Disease: Improving Global Outcomes
PRISMA: Preferred Reporting Items for Systematic Reviews and Meta-analyses
RCT: randomized controlled trial
KRT: kidney replacement therapy
LOS: length of stay
CI: confidence interval
RR: risk ratio
OR: odds ratio
