# Supplementary material for: Impact of AKI care bundles on kidney and patient outcomes in hospitalized patients: a systematic review and meta-analysis
Source: BMC Nephrol. 2021 Oct 8;22:335. doi: 10.1186/s12882-021-02534-4 (PMC8501614; doi:10.1186/s12882-021-02534-4)

**Figure S1: Occurence of moderate-severe AKI and KRT (AKI episodes)**

Figure S1a: Occurrence of moderate-severe AKI


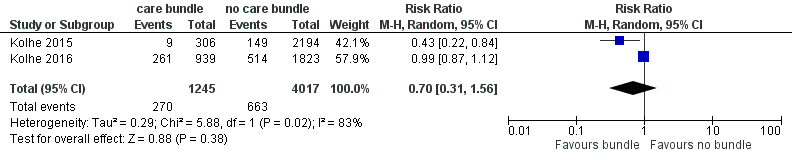


Figure S1b: Occurrence of KRT in patients with AKI


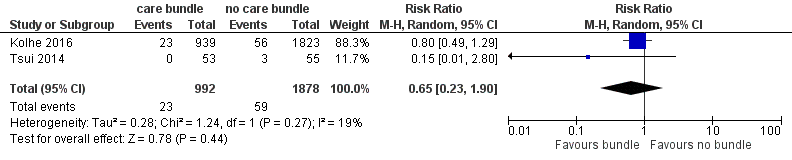


**Figure S2: Outcomes in ICU patients only**

Figure S2a: AKI occurrence


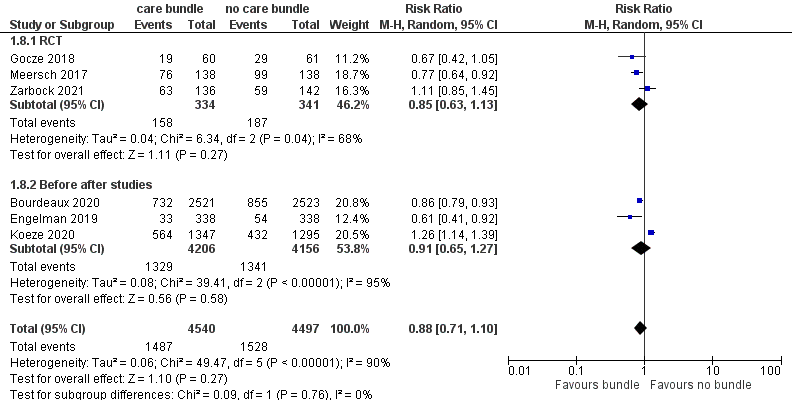


Figure S2b: Occurrence of moderate-severe AKI


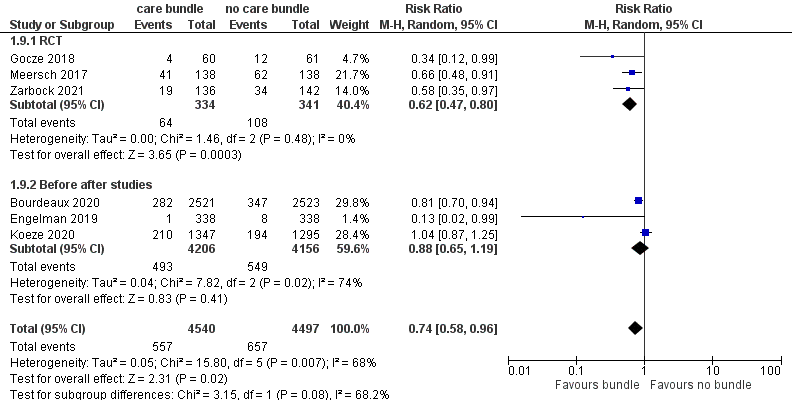


Figure S2c: Use of KRT


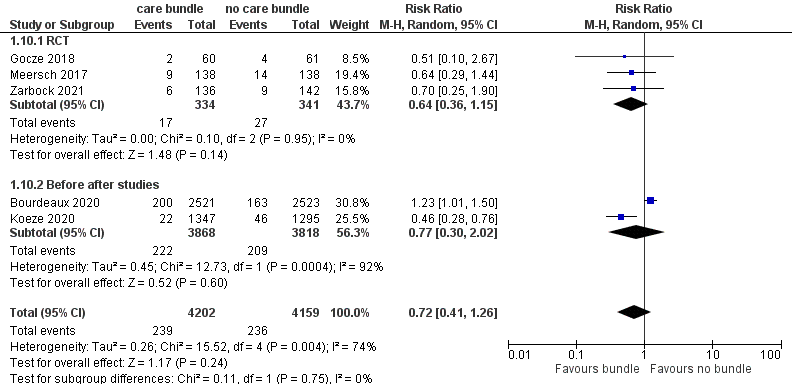


**Figure S3: mortality outcomes**

Figure S3a: ICU mortality with and without AKI care bundle (AKI episodes)


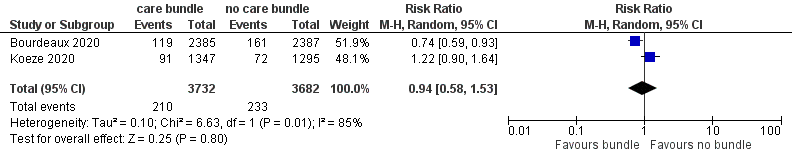


Figure S3b: 30 day mortality with and without AKI care bundle (AKI episodes)


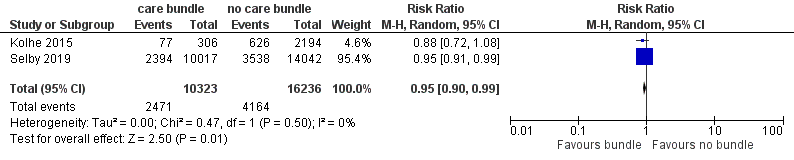


Figure S3c: 30 day mortality with and without AKI care bundle (all patients)


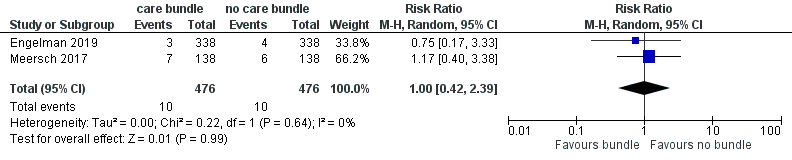


**Figure S4: Hospital length-of-stay with and without AKI care bundle (AKI episodes)**


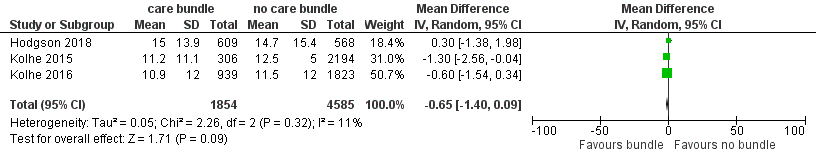

Supplement: Supplementary file 1 — Additional file 1: Figure S1: Occurrence of moderate-severe AKI and KRT (AKI episodes). Figure S2: Occurrence of AKI, moderate-severe AKI and KRT in ICU patients only. Figure S3: ICU and 30 day mortality. Figure S4: Hospital length-of-stay (AKI episodes). [file 12882_2021_2534_MOESM1_ESM.docx]
