# Supplementary material for: Impact of AKI care bundles on kidney and patient outcomes in hospitalized patients: a systematic review and meta-analysis
Source: BMC Nephrol. 2021 Oct 8;22:335. doi: 10.1186/s12882-021-02534-4 (PMC8501614; doi:10.1186/s12882-021-02534-4)

**Figure S5: Funnel plots of main outcomes**

Figure a: AKI occurrence


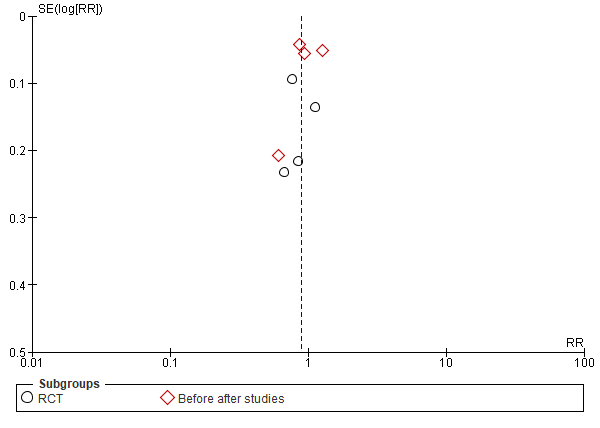


Figure b: Occurrence of moderate severe AKI


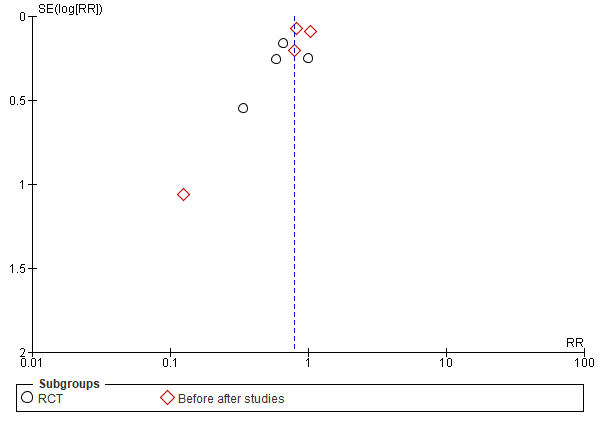


Figure c: Use of KRT


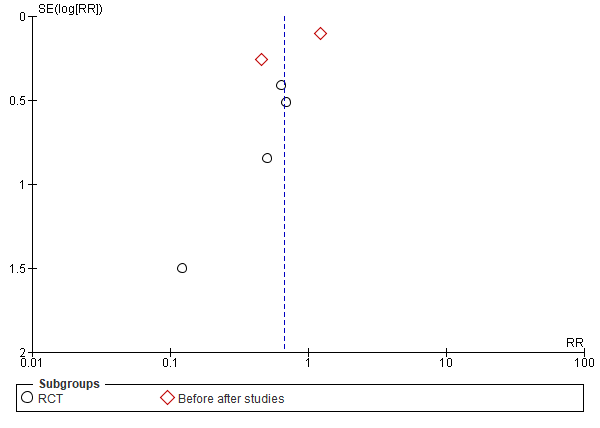


Figure d: In-hospital mortality (all pts)


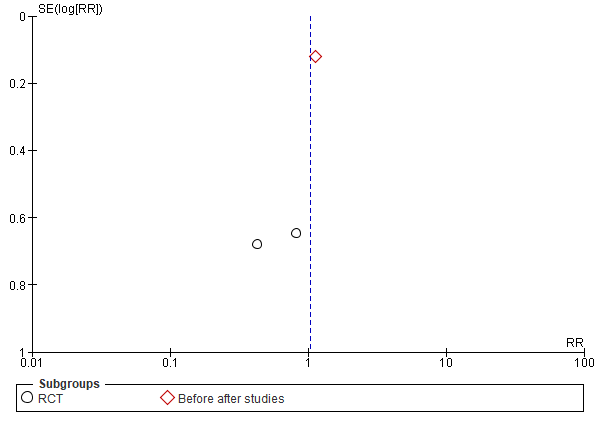


Figure e: In-hospital mortality (AKI pts)


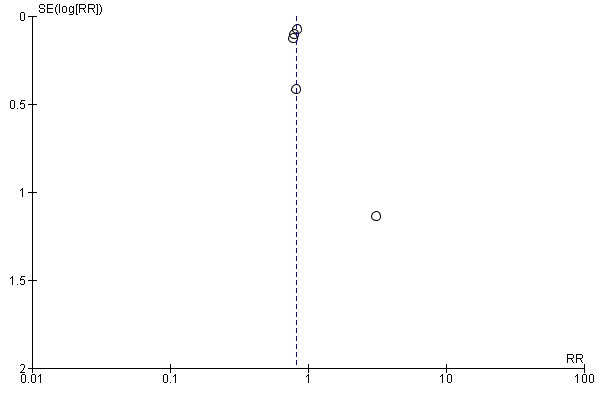

Supplement: Supplementary file 2 — Additional file 2: Figure S5: Funnel plots of main outcomes. [file 12882_2021_2534_MOESM2_ESM.docx]
