# Supplementary material for: Impact of AKI care bundles on kidney and patient outcomes in hospitalized patients: a systematic review and meta-analysis
Source: BMC Nephrol. 2021 Oct 8;22:335. doi: 10.1186/s12882-021-02534-4 (PMC8501614; doi:10.1186/s12882-021-02534-4)
Supplement: Supplementary file 4 — Additional file 4: Table TS2: Risk of bias tool for non-randomized trials. [file 12882_2021_2534_MOESM4_ESM.docx]

Table S2: Risk of bias analysis of intervention studies (<https://www.nhlbi.nih.gov/health-topics/study-quality-assessment-tools>)

| 1. Was the study question or objective clearly stated? |
| --- |
| 2. Were eligibility/selection criteria for the study population prespecified and clearly described? |
| 3. Were the participants in the study representative of those who would be eligible for the test/service/intervention in the general or clinical population of interest? |
| 4. Were all eligible participants that met the prespecified entry criteria enrolled? |
| 5. Was the sample size sufficiently large to provide confidence in the findings? |
| 6. Was the test/service/intervention clearly described and delivered consistently across the study population? |
| 7. Were the outcome measures prespecified, clearly defined, valid, reliable, and assessed consistently across all study participants? |
| 8. Were the people assessing the outcomes blinded to the participants' exposures/interventions? |
| 9. Was the loss to follow-up after baseline 20% or less? Were those lost to follow-up accounted for in the analysis? |
| 10. Did the statistical methods examine changes in outcome measures from before to after the intervention? Were statistical tests done that provided p values for the pre-to-post changes? |
| 11. Were outcome measures of interest taken multiple times before the intervention and multiple times after the intervention (i.e., did they use an interrupted time-series design)? |
| 12. If the intervention was conducted at a group level (e.g., a whole hospital, a community, etc.) did the statistical analysis take into account the use of individual-level data to determine effects at the group level? |
