# Supplementary material for: Impact of AKI care bundles on kidney and patient outcomes in hospitalized patients: a systematic review and meta-analysis
Source: BMC Nephrol. 2021 Oct 8;22:335. doi: 10.1186/s12882-021-02534-4 (PMC8501614; doi:10.1186/s12882-021-02534-4)
Supplement: Supplementary file 5 — Additional file 5: Table TS3: Risk of bias analysis. [file 12882_2021_2534_MOESM5_ESM.docx]

**Table S3: Risk of bias analysis**

| B-A studies  Questions | Bourdeaux 2020 | Engelman  2019 | Hodsgon  2018 | Joslin  2015 | Koeze  2020 | Kolhe  2015 | Kolhe  2016 | Selby  2019 | Tsui  2014 |
| --- | --- | --- | --- | --- | --- | --- | --- | --- | --- |
| 1 objectives |  |  |  |  |  |  |  |  |  |
| 2 selection |  |  |  |  |  |  |  |  |  |
| 3 representative |  |  |  |  |  |  |  |  |  |
| 4 enrollment |  |  |  |  |  |  |  |  |  |
| 5 sample size |  |  |  |  |  |  |  |  |  |
| 6 intervention |  |  |  |  |  |  |  |  |  |
| 7 outcomes |  |  |  |  |  |  |  |  |  |
| 8 blinded |  |  |  |  |  |  |  |  |  |
| 9 loss follow-up |  |  |  |  |  |  |  |  |  |
| 10 statistics |  |  |  |  |  |  |  |  |  |
| 11 outcomes multiple times |  |  |  |  |  |  |  |  |  |
| 12 group/individual |  |  |  |  |  |  |  |  |  |

| RCT  Bias | **Gocze**  **2018** | **Meersch**  **2017** | **Schanz**  **2018** | **Zarbock**  **2021** |
| --- | --- | --- | --- | --- |
| Random sequence generation  (selection bias) |  |  |  |  |
| Allocation concealment  (selection bias) |  |  |  |  |
| Blinding of participants and personnel  (performance bias) |  |  |  |  |
| Blinding of outcome assessment  (detection bias) |  |  |  |  |
| Incomplete outcome data  (attrition bias) |  |  |  |  |
| Selective reporting  (reporting bias) |  |  |  |  |
